# Supplementary material for: Conditional chemoconnectomics (cCCTomics) as a strategy for efficient and conditional targeting of chemical transmission
Source: eLife. 2024 Apr 30;12:RP91927. doi: 10.7554/eLife.91927 (PMC11060718; doi:10.7554/eLife.91927)
Supplement: Figure 3—source data 3. [file elife-91927-fig3-data3.zip › Figure 3 source data3 part 1.zip!/Figure 3-Source data 3-part1/1063-13-CRISPResso_Report_8cz_fsdr.zip!/CRISPResso_on_1063_16/9.Alleles_frequency_table_around_sgRNA_cgtcgagcacatgacatcaa.pdf]

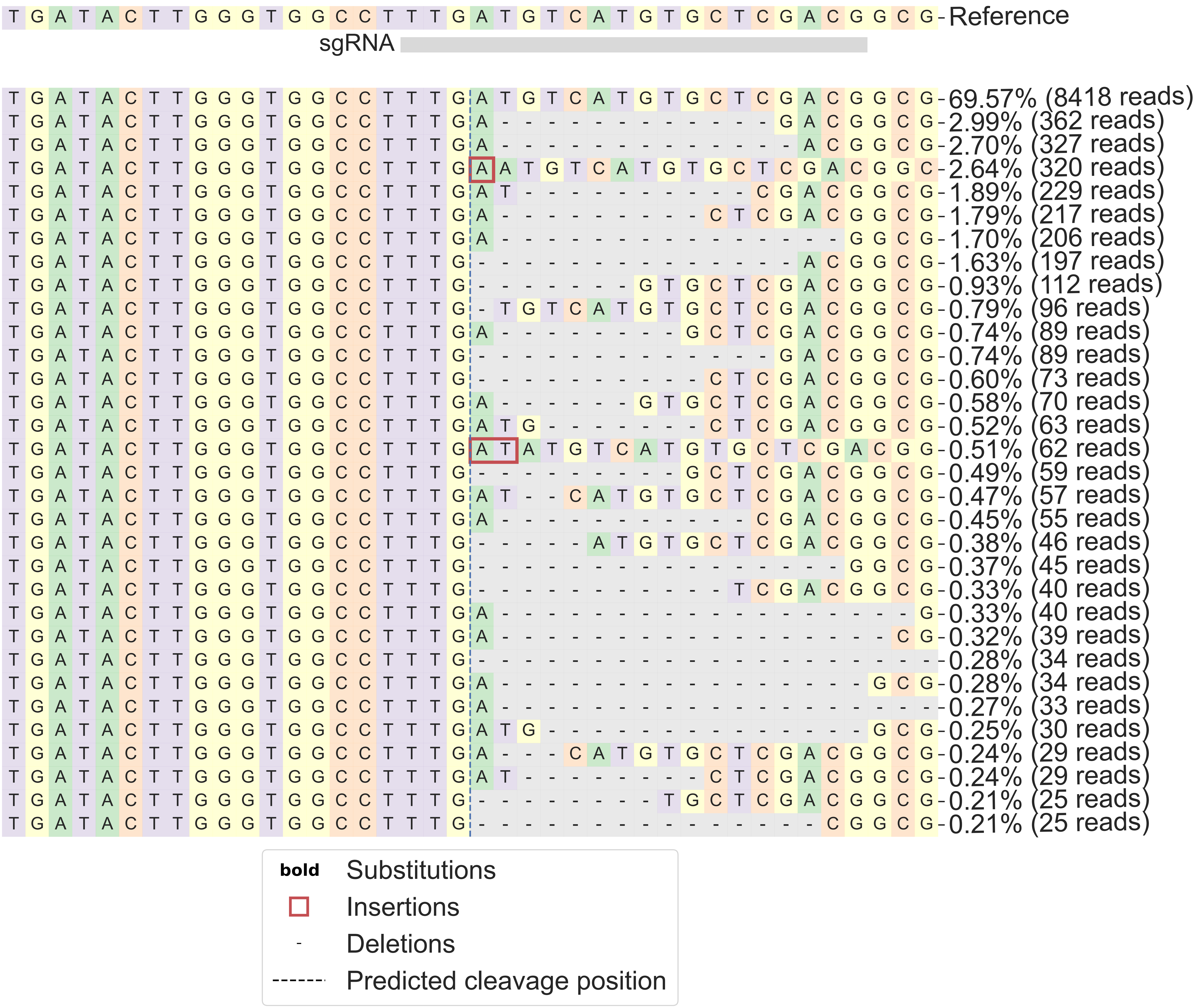
T G A T A C T T G G G T G G C C T T T G A T G T C A T G T G C T C G A C G G C G
Reference
sgRNA
69.57% (8418 reads)
2.99% (362 reads)
2.70% (327 reads)
2.64% (320 reads)
1.89% (229 reads)
1.79% (217 reads)
1.70% (206 reads)
1.63% (197 reads)
0.93% (112 reads)
0.79% (96 reads)
0.74% (89 reads)
0.74% (89 reads)
0.60% (73 reads)
0.58% (70 reads)
0.52% (63 reads)
0.51% (62 reads)
0.49% (59 reads)
0.47% (57 reads)
0.45% (55 reads)
0.38% (46 reads)
0.37% (45 reads)
0.33% (40 reads)
0.33% (40 reads)
0.32% (39 reads)
0.28% (34 reads)
0.28% (34 reads)
0.27% (33 reads)
0.25% (30 reads)
0.24% (29 reads)
0.24% (29 reads)
0.21% (25 reads)
0.21% (25 reads)
bold Substitutions
Insertions
Deletions
Predicted cleavage position
